# High ERCC1 expression predicts cisplatin-based chemotherapy resistance and poor outcome in unresectable squamous cell carcinoma of head and neck in a betel-chewing area

**DOI:** 10.1186/1479-5876-9-31

**Published:** 2011-03-23

**Authors:** Tai-Jan Chiu, Chang-Han Chen, Chih-Yen Chien, Shau-Hsuan Li, Hsin-Ting Tsai, Yi-Ju Chen

**Affiliations:** 1 Department of Medical Oncology, Chang Gung Memorial Hospital-Kaohsiung Medical Center, Chang Gung University, College of Medicine, Kaohsiung, Taiwan; 2 Department of Otolaryngology, Chang Gung Memorial Hospital-Kaohsiung Medical Center, Chang Gung University College of Medicine, Kaohsiung, Taiwan; 3 Department of Pathology, E-Da hospital, Kaohsiung, Taiwan; 4 Kaohsiung Chang Gung Head and Neck Oncology Group, Cancer Center, Chang Gung Memorial Hospital-Kaohsiung Medical Center, Kaohsiung, Taiwan; 5 Center for Translational Research in Biomedical Sciences, Chang Gung Memorial Hospital-Kaohsiung Medical Center; 6 Institute of Clinical Medical Sciences, Chang Gung University, Kaohsiung, Taiwan

**Keywords:** ERCC1, squamous cell carcinoma of head and neck, betel nuts, induction chemotherapy, chemoradiation

## Abstract

**Background:**

This study was to evaluate the effect of excision repair cross-complementation group 1(ERCC1) expression on response to cisplatin-based induction chemotherapy (IC) followed by concurrent chemoradiation (CCRT) in locally advanced unresectable head and neck squamous cell carcinoma (HNSCC) patients.

**Methods:**

Fifty-seven patients with locally advanced unresectable HNSCC who received cisplatin-based IC followed by CCRT from January 1, 2006 through January 1, 2008. Eligibility criteria included presence of biopsy-proven HNSCC without a prior history of chemotherapy or radiotherapy. Immunohistochemistry was used to assess ERCC1 expression in pretreatment biopsy specimens from paraffin blocks. Clinical parameters, including smoking, alcohol consumption and betel nuts chewing, were obtained from the medical records.

**Results:**

The 12-month progression-free survival (PFS) and 2-year overall survival (OS) rates of fifty-seven patients were 61.1% and 61.0%, respectively. Among these patients, thirty-one patients had low ERCC1 expression and forty-one patients responded to IC followed by CCRT. Univariate analyses showed that patients with low expression of ERCC1 had a significantly higher 12-month PFS rates (73.3% vs. 42.3%, p < 0.001) and 2-year OS (74.2 vs. 44.4%, p = 0.023) rates. Multivariate analysis showed that for patients who did not chew betel nuts and had low expression of ERCC1 were independent predictors for prolonged survival.

**Conclusions:**

Our study suggest that a high expression of ERCC1 predict a poor response and survival to cisplatin-based IC followed by CCRT in patients with locally advanced unresectable HNSCC in betel nut chewing area.

## Background

Squamous cell carcinoma of the head and neck (HNSCC) is the sixth most common cancer in the world [1] and two-thirds of these patients initially present with locally advanced disease [2]. In Taiwan, HNSCC rates 4^th ^in male cancer-related deaths [3] among middle-aged male patients between 25 and 45 years old [4]. Most HNSCC patients in Taiwan diagnosed with advanced disease are young men. The main risk factors of this unique patient population are the habitual consumption of cigarettes, alcohol, and betel nuts [5,6].

Although patients with locally advanced HNSCC receive surgery and radiotherapy, less than 30% will be cured, and locoregional recurrences or distant metastases develop in 40% to 60% patients [7,8], which occurs with a median survival rate of no more than 6 months [9]. Some studies have demonstrated improved locoregional control and overall survival by adding chemotherapy to radiotherapy concurrently [10]. The Meta-Analysis of Chemotherapy in Head and Neck Cancer (MACH-NC) study showed that concomitant chemoradiation is superior to RT alone for patients with advanced HNSCC and chemoradiotherapy (radiotherapy plus concurrent chemotherapy) has become the standard of care for patients with unresectable HNSCC [11,12]. However, the best chemotherapeutic regimen combined with RT in HNSCC has yet to be defined; the concomitant administration of cisplatin represents a widely accepted choice. It has been reported that induction chemotherapy (IC) with cisplatin and fluorouracil (PF) benefits this disease [12-14] and results in a significantly improved 5-year survival rate in patients with locally advanced disease compared to surgery and standard radiotherapy alone [12].

In Taiwan, for public healthy insurance, cisplatin is the backbone of the chemotherapy regimen as a component of IC and CCRT in the treatment of locally advanced HNSCC. Its main cytotoxic activity is based on the formation of DNA adducts, which cause inter- and intrastrand cross-linking. These DNA cross-links are recognized and removed by the nucleotide excision repair pathway which arms to guard the integrity of the genome [15,16]. The enzyme excision repair cross-complementation group 1(ERCC1) plays a rate limiting role in the nucleotide excision repair pathway, and its expression has been associated with survival in patients with various malignancies [17-19]. The relation between ERCC1 expression and resistance to platinum compounds had been found by some clinical studies in patients with advanced-stage gastric, ovarian, colorectal, esophageal, and non-small-cell lung cancers [15,17,19-21]. However, there are only few studies to elucidate the relationship between ERCC1 expression and prognosis in patients with locally advanced HNSCC treated with CCRT. The purpose of this study was to evaluate whether the immunohistochemical expression status of ERCC1 can predict the treatment response and survival in patients with unresectable HNSCC being treated with cisplatin-based IC followed by CCRT.

## Methods

### Patients and treatment

A total of 57 patients with pathologically proven locally advanced inoperable HNSCC were treated with IC followed by CCRT between January 1, 2006 and January 1, 2008 at Kaohsiung Chang-Gung Medical Center (Taiwan). To be included, all the patients had to have a biopsy-proven previously untreated IV (M0) unresectable squamous cell carcinoma of the head and neck region, have no synchronous primary tumors, and be ≥18 years old. In addition, the patients had to have a performance status (PS) of ≤2 on the Eastern Cooperative Oncology Group (ECOG) scale, adequate bone marrow, hepatic and renal function (creatinine clearance >60 ml/min), and a computed tomography or magnetic resonance image scan of the head and neck region within three weeks prior to the initiation of treatment. The clinicopathological information including age, gender, tumor (T) stage, nodal (N) status, TNM stage, and survival was obtained from the clinical records. The histories of betel nuts chewing, alcohol and tobacco use were obtained by our detailed questioning at the patients' first visit to the otolaryngology clinic of the hospital.

The IC consisted of 2 cycles of cisplatin 75 mg/m2 and fluorouracil (5-FU) (1000 mg/m2) given as a continuous 24-h infusion for four days. The two cycles of IC were administered every four weeks. After IC, all patients received CCRT. During the CCRT, cisplatin was administered weekly at a dose of 40 mg/m2. RT was delivered 3-4 weeks after the completion of the IC with a linear accelerator. Ondansentron ± dexamethasone was used as antiemetic treatment. The response to IC followed by CCRT was assessed according to the World Health Organization (WHO) criteria. Surgery was performed six to twelve weeks after completion of IC followed by CCRT regimen for patients who had residual disease. Surgery was also allowed for patients who did not complete chemoradiation and had resectable residual disease at the primary site or in the neck. Patients were evaluated by CT scan or MRI of the head and neck every three months. Informed consent was obtained from study participants and protocol for this study was approved by the Institutional Review Boards of Chang-Gung Medical Center (Taiwan).

### Immunohistochemical staining for ERCC1

Adjacent non-cancerous and tumor HNSCC tissue samples were selected by a pathologist based on diagnosis and microscopic morphology. Adjacent non-cancerous tissue and tumor tissues were fixed with 10% buffered formalin embedded in paraffin and decalcified in 10% EDTA solution. Representative blocks of the formalin-fixed, paraffin-embedded tissues were cut to 4 mm and deparaffinized with xylene and rehydrated in a series of ethanol washes (100, 90, 80, and 70%). Slides were washed with phosphate-buffered saline (PBS) and treated with 3% H_2_O_2 _for 30 minutes to block endogenous peroxidase activity. Next, the sections were microwaved in 10 mM citrate buffer, pH 6.0, to unmask the epitopes. After antigen retrieval, the sections were incubated with diluted anti-ERCC1 antibody (monoclonal; 8F1; Thermo scientific, Fremont, CA, USA; 1:100), for 3 h followed by washing with PBS. Horseradish peroxidase/Fab polymer conjugate (PicTure™-Plus kit; Zymed, South San Francisco, CA, USA) was then applied to the sections for 30 min followed by washing with PBS. Finally, the sections were incubated with diaminobenzidine for 5 min to develop the signals. A negative control was run simultaneously by omitting the primary antibody.

### Evaluation of ERCC1 expression

Two pathologists, who were unaware of the clinical data, evaluated the ERCC1 staining independently under a light microscope at a magnification of × 400. The pathologists recorded whether tumor or stromal cells expressed ERCC1. The staining intensity was graded on a scale of 0-3, using adjacent nonmalignant cells as a reference (intensity 2). Five images of representative areas were acquired for each specimen. The percentage of positive nuclei was calculated for each specimen, and a proportion score was assigned (0 if 0%, 0.1 if 1-9%, 0.5 if 10-49%, and 1.0 if ≧ 50%). The proportion score was multiplied by the staining intensity to obtain a final semi-quantitative H score. The median value of the H score was chosen as the cutoff point for separating low and high levels of ERCC1 expression [22].

### Statistical analysis

Statistical analyses of 2 × 2 tables of categorical variables were performed using Pearson's *x^2 ^*test or Fisher's exact test, where appropriate. Survival probability analyses were performed using the Kaplan-Meier method. Survival was calculated from the date of start of chemotherapy to the date of death or most recent follow-up. Progression free survival (PFS) was defined as the time from the date of first chemotherapy to the date of first observation of disease progression, or relapse, or death due to any cause. Significance between group differences was assessed by the log-rank test. Multivariate analyses were performed using a logistic regression model for response and Cox regression models for PFS and overall survival (OS). Factors with p-values < 0.05 in univariate analyses were examined with multivariate regression models. All statistical tests were two-sided, with significance defined as *p *< 0.05. Analyses were performed using SPSS version 13.

## Result

### Patient characteristics

The median age of the patients was 53 years (range 36-72 years), and fifty-five (96.5%) out of 57 were men. Ten patients had IVA and 47 had stage IVB disease. The most common sites were the oral cavity (24/57, 42.1%), followed by the oropharynx (21/57, 36.8%) (Table 1). The median radiation they received was 6600 cGy. Nineteen patients received more than 70 Gy of radiation dose. All patients had received their IC and 53 patients completed the followed up CCRT.

**Table 1:** Correlation between expression of ERCC1 and clinicopathological factors of HNSCC

		ERCC1		P	Multivariates analysis	P
	No. of patients	Low expression	High expression		OR (95% CI)	
Age						
≦50	22 (38.6%)	11 (50.0%)	11 (50.0%)	0.598	1	
> 50	35 (61.4%)	20 (57.1%)	15 (42.9%)		0.47 (0.11, 2.11)	0315
Gender						
Male	55 (96.5%)	30 (54.5%)	25 (45.5%)	1.000	1	
Female	2 (3.5%)	1 (50%)	1 (50%)		48.36 (0.54, 4313.32)	0.090
Tumor Site						
oral cavity	24 (42.1%)	11 (45.8%)	13 (54.2%)	0.057	1	
oropharynx	21 (36.8%)	10 (47.6%)	11 (52.4%)		1.58 (0.35, 7.07)	0.549
hypopharynx/Larynx	12 (21.1%)	10 (83.3%)	2 (16.7%)		0.096 (0.007, 1.33)	0.081
Stage						
IVa	10 (17.5%)	7 (70.0%)	3 (30.0%)	0.319	1	
IVb	47(82.5%)	24 (51.1%)	23 (48.9%)		3.33 (0.39, 27.89)	0.276
T stage						
1/2	6 (10.5%)	6 (100%)	0 (0%)	0.027*	1	
3/4	51 (89.5%)	25 (49.0%)	26 (51.0%)		Indeterminate	0.999
N stage						
negative	12 (21.1%)	5 (41.7%)	7 (58.3%)	0.503	1	
positive	45 (78.9%)	26 (57.8%)	19 (42.2%)		0.75 (0.15, 3.64)	0.727
Alcohol drinking						
Never	11 (17.2%)	4 (36.4%)	7 (63.6%)	0.318	1	
Yes	46 (82.8%)	27 (58.7%)	19 (41.3%)		2.49 (0.31, 19.88)	0.388
Smoking						
Never	9 (15.8%)	3 (33.3%)	6 (66.7%)	0.275	1	
Yes	48 (84.2%)	28 (58.3%)	20 (41.7%)		1.77 (010, 30.72)	0.695
Betel nuts						
Never	20 (35.1%)	8 (40.0%)	12 (60.0%)	0.109	1	
Yes	37 (64.9%)	23 (62.2%)	14 (37.8%)		12.78 (1.28-127.62)	0.030*

### Clinico-pathologic factors of HNSCC patients with ERCC1 expression

**Figure 1:**
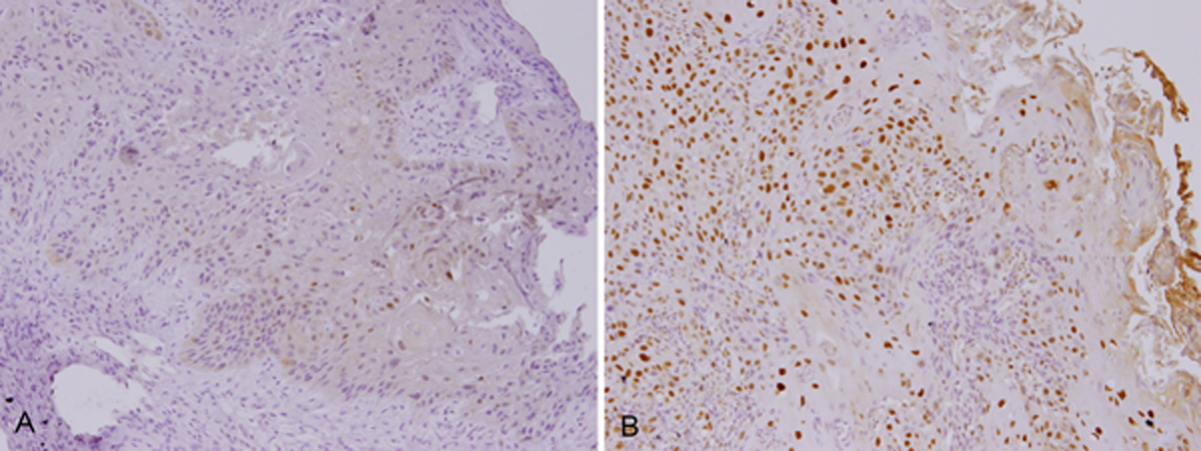
**Analysis of ERCC1 expression in head and neck squamous cell carcinoma**. ERCC1 expression was determined using immunohistochemistry. A) Low ERCC1 expression (200× magnification). B) High ERCC1 expression (200× magnification).

To investigate whether the increased expression of ERCC1 was associated with various prognostic factors, such as age, gender, and TNM pathologic classification, we classified the patients into two groups based on their immunohistochemical results (low *vs*. high ERCC1 expression) (Figure 1A and 1B). The median H score for HNSCC was 1.5. Twenty-six (46%) tumors had an H score of more than 1.5 and were thus defined as having a high expression of ERCC1. As can be seen in Table 1 a summary of result of the ERCC1 immunostaining of the cancer cells and its correlation with the clinicopathologic variables, the high and low ERCC1 expression groups did not **s**ignificantly with regard to age, gender, TNM tumor stage, and node metastatic status, alcohol drinking or smoking (Table 1). The high ERCC1 expression group had a higher T stage (T3-4) (*p = 0.027*). Those with squamous cell carcinoma of the hypopharynx/larynx were found to have marginal lower expression of ERCC1. Interestingly, in our multivariate regression model, patients who habitually chewed betel nuts had a significantly higher expression of ERCC1.

### Relationship between treatment response and ERCC1 expression

The overall response rate after CCRT for all patients was 72% (41/57 with 28 complete responses and 13 partial responses; 9 had stable disease and 7 progressive disease). Patients with low expression of ERCC1 had a higher treatment response (28/31, 90.3%) than the high expression group (13/26, 50%) (*p = 0.002*, Table 2).

**Table 2:** Relationship between treatment response and clinicopathological factors

	Treatment response			Multi-variates	
			
	CR/PR	SD/PD	P	OR (95%CI)	P
Age					
≦50	15 (68.2%)	7 (31.8%)	0.844	1	
> 50	26 (74.3%)	9 (25.7%)		0.53 (0.07, 3.65)	0.520
Gender					
male	39 (70.9%)	16 (29.1%)	1.000	1	
female	2 (100%)	0 (0%)		Indeterminate	1.000
Tumor Site					
oral cavity	15 (62.5%)	9 (37.5%)	0.049*	1	
oropharynx	14 (66.7%)	7 (33.3%)		1.29 (0.21, 7.70)	0.778
hypopharynx/larynx	12 (100%)	0		Indeterminate	0.998
Stage					
IVa	10 (100%)	0 (0%)	0.048*	1	
IVb	31 (66.0%)	16 (34.0%)		Indeterminate	0.998
T stage					
1/2	6 (100%)	0 (0%)	0.170	1	
3/4	35 (68.6%)	16 (31.4%)		Indeterminate	0.999
N stage					
negative	8 (66.7%)	4 (25.0%)	0.732	1	
positive	33 (73.3%)	12 (26.7%)		0.75 (0.06, 8.54)	0.818
Radiation					
≦6000 cGy	14 (70.0%)	6 (30.0%)	1.000	1	
> 6000 cGy	27 (75.7%)	10 (24.3%)		0.22 (0.02, 2.43)	0.222
Alcohol drinking					
never	9 (81.8%)	2 (18.2%)	0.710	1	0.120
yes	32 (69.6%)	14 (30.4%)		0.08 (0.004, 1.89)	
Smoking					
never	7 (77.8%)	2 (22.2%)	1.000	1	
yes	34 (70.8%)	14 (29.2%)		2.97 (0.10, 86.59)	0.526
Betel nuts					
never	16 (80.0%)	4 (20.0%)	0.491	1	
yes	25 (67.6%)	12 (32.4%)		0.38 (0.03, 4.55)	0.452
ERCC1					
low expression	28 (90.3%)	3 (9.7%)	0.002*	1	
high expression	13 (20.0%)	13 (50.0%)		0.07 (0.009, 055).	0.012*

CR, complete response; PR, partial response; SD, stable disease; PD, disease progression; RTO, radiotherapy; OR, odds ratio; CI, confidence interval

### Relationship between survival and ERCC1 expression

**Figure 2:**
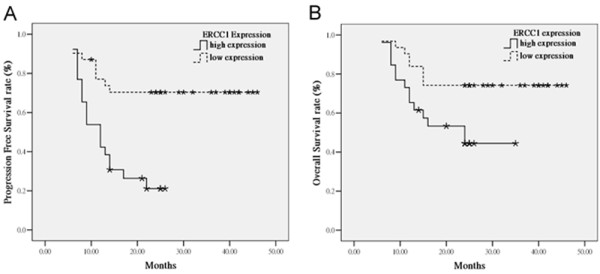
**Kaplan-Meier estimates of the probability of survival**. **(A) **PFS according to ERCC1 expression. PFS: progression free survival. **(B) **OS according to ERCC1 expression. OS: overall survival

The median follow-up was 24.0 months (6 - 46 months). The overall 12-month PFS rate was 61.1% and the 2-year OS rate was 61.0%. The 12-month PFS for patients with low expression of ERCC1 was 73.3% compared with 42.3% for patients with high expression of ERCC1 (p < 0.001, Figure 2A). The 2-year OS rate was significantly higher in patients with low expression of ERCC1 (74.2%) than in those with high expression of ERCC1 (44.4%) (P = 0.023, Figure 2B). Univariate analysis showed that tumor stage and tumor location were important factors affecting the OS and PFS (Table 3), though ERCC1 expression and betel nuts chewing were the prognostic factors in OS by multivariate analysis according to Cox regression model (Table 4).

**Table 3:** Univariate analyses of prognostic factors for survival

Variables	No. of patients	Cumulative 12-month preogresion free survival rate	P	Cumulative 2-year overall survival rate	*P*
**Age**					
< 50	22	49.0%	0.725	50.0%	0.152
≧50	35	68.6%		68.0%	
**Gender**					
male	55	61.5%	0.878	65.2%	0.553
female	2	50.0%		50.0%	
**Site**					
oral cavity	24	41.7%		45.5%	0.093
oropharynx	21	66.7%	0.101	66.6%	
Hypopharynx/larynx	12	81.8%	0.035*	83.3%	
**Stage**					
IVa	10	80.0%	0.119	90.0%	0.049*
IVb	47	57.2%		54.7%	
**T stage**					
0-2	6	66.7%	0.396	66.7%	0.694
3-4	51	60.5%		60.3%	
**N stage**					
negative	12	50.0%	0.837	72.9%	0.350
positive	45	57.3%		57.8%	
**Radiation**					
≦6000 cGy	20	55.0%	0.304	55.0%	0.412
> 6000 cGy	37	64.5%		64.1%	
**Alcohol**					
never	11	63.6%	1.000	63.6%	0.754
yes	46	60.6%		64.8%	
**Smoking**					
never	9	66.7%	0.614	66.7%	0.679
yes	48	60.1%		60.0%	
**Betel nuts**					
never	20	70.0%	0.638	74.0%	0.123
yes	37	56.3%		54.1%	
**ERCC1**					
low expression	31	73.7%	< 0.001*	74.2%	0.023*
high expression	26	42.3%		44.4%	

**Table 4:** Risk factors affecting 1-year disease free survival and 2-year overall survival rate determined by Cox regression analysis

Variables	PFS		OS	
				
	HR (95%CI)	*P*	HR (95%CI)	*P*
**ERCC1 expression**				
Low vs High	0.27 (0.12-0.61)	0.001	0.31 (0.13-0.75)	0.010
**Betel nuts**				
Never vs Used	NE	0.647	0.35 (0.13-0.98)	0.045

CI, confidence interval; HR, Hazard ratio

## Discussion

It is of special interest that in our study that specimens from patients who habitually chewed betel nuts had high expression of ERCC1. Betel nut chewing is a common habit among those who live in South Asia, including Taiwan [23], and is known as one cause of HNSCC [24]. There are many compounds in the betel nut that have been correlated with carcinogenesis; the habit of chewing betel nut is related to persistent damage of the oral mucosa as well as precancerous lesions such as leukoplakia and erythroplakia, and oral submucosal fibrosis [25]. In previous reports, overexpression of epidermal growth factor receptor (EGFR) was found to be involved in betel nut-related HNSCC [26,27]. However, the relationship between betel nut and ERCC1 expression has not been reported before. In this study, we find tissue samples from patients with habitual consumption of betel nuts showed significant correlation with high ERCC1 expression. This finding awaits confirmation by prospective studies with large numbers of patients.

In this study, Forty-six percent of the patients with inoperable HNSCC had a high expression of ERCC1. Patients with a high expression of ERCC1 had a lower treatment response rate to IC followed by CCRT than those with low expression of ERCC1. In addition, low ERCC1 expression was associated with a significantly longer PFS and OS. Multivariate analysis revealed that low expression of ERCC1 to be an independent factor associated with a lower risk of cancer death (HR 0.31, *p *= 0.010). Our findings are consistent with previous report of an increase in tumor response and prolongation of OS in patients treated by cisplatin based IC followed by CCRT for locally advanced HNSCC [28-30]. Moreover, the relationship between the expression of ERCC1 and tumor response or survival has also been demonstrated in esophageal cancer patients treated with chemoradiotherapy [31] and non-small cell lung cancer treated with cisplatin-based adjuvant chemotherapy [22].

However, in patients with locally advanced HNSCC treated with cetuximab-based CCRT, ERCC1 expression has not been found to predict treatment response [32]. In this context, we assume that pre-therapeutic ERCC1 protein levels within tumor cells might be correlated with their cisplatin-related DNA damage repair capacity. A less efficient DNA-repair capacity could affect the cellular response to DNA damage and could thus render cancer cells more sensitive to cisplatin. In addition, Nix et al. has reported an association between both ERCC1 and XRCC1 and radioresistance in laryngeal tumors [33].

Cetuximab is an IgG1 monoclonal antibody against the ligand-binding domain of EGFR. Cetuximab binds EGFR, sequesters the receptor in the cytoplasm and eventually targets it for degradation. In vitro studies have demonstrated that this antibody enhances the radio-sensitivity in HNSCC cells [34,35] through several processes, such as DNAPK, which are reviewed in Mukesh et al. [36]. When cetuximab is combined with radiation, it has been found to inhibit the nuclear translocation of the complex between DNA-dependent protein kinase and EGFR and then delayed the DNA repair [37-39]. Oxaliplatin induced double-strand breaks [40]. When cetuximab was combined with oxaliplatin, cetuximab reduced the expression of ERCC-1 and other genes involved in DNA replication initiation [41,42]. We might find a subgroup of patients with high ERCC1 expression having poor response to cisplatin-based IC and CCRT that is particularly benefited from treatments with cetuximab and other chemotherapeutic agents.

Our study has several limitations. First, the study was based on a retrospective analysis and only there were only 57 patients accumulated over a short period. The primary tumor site was also heterogeneous, and the prognosis of HNSCC is dependent on the primary tumor site. In our study, those oral cavity cancer had the worst prognosis and laryngeal cancer a good prognosis, although we found no significant difference in our multi-variate analyses. Second, some patients with IC followed by CCRT had a partial response and received further salvage surgery. Patients who receive salvage surgery had significantly longer PFS and OS rates than those who did not receive such surgery. The salvage surgery may affect the relationship between ERCC1 expression and survival. It also suggested that those patients with lower expression of ERCC1 would benefit from the potential downstage by our treatment protocol and become resectable. Our study was comprised only a small number of patients for each tumor location, and so we may need more homogeneous and a larger number of patients to validate this finding.

## Conclusion

This present study suggests that ERCC1 mediated repair of DNA damage contributes to the clinical outcome in patients with locally advanced inoperable HNSCC treated with cisplatin-based IC and CCRT. In this context, it is strongly recommended that tissue be collected to assess ERCC1 expression before cisplatin-based induction chemotherapy and concurrent chemoradiotherapy. If patients with habit of betel nuts chewing may have higher chance of high ERCC1 expression, they should consider other treatment approach modalities.

## Competing interests

The authors declare that they have no competing interests.

## Authors' contributions

TJC and CHC conceived the study design, carried out and coordinated immunohistochemical examinations of tumor specimens and data analysis, and drafted the manuscript. CYC and HTT participated in the interpretation of data and conducted immunohistochemistry analysis. SHL collected the clinical data of patients and performed statistical data analysis. YJC coordinated the study and were involved in drafting the manuscript and revised it critically. All authors read and approved the final manuscript.
